# Supplementary material for: The effect of end-of-life decision-making tools on patient and family-related outcomes of care among ethnocultural minorities: A systematic review
Source: PLoS One. 2022 Aug 4;17(8):e0272436. doi: 10.1371/journal.pone.0272436 (PMC9352046; doi:10.1371/journal.pone.0272436)
Supplement: S1 Appendix — (DOCX) [file pone.0272436.s001.docx]

**S1 Appendix. Search strategy for electronic databases.**

**Medline:**

1. decision making/ or choice behavior/ or consensus/ or "dissent and disputes"/ or negotiating/

2. patient care planning/ or advance care planning/ or advance directives/ or living wills/ or critical pathways/ or patient-centered care/ or informed consent/

3. Decision Support Systems, Clinical/ or Decision Support Techniques/

4. ((plan* or decision*) adj4 (care or medical or treatment)).tw,kf.

5. (goal* adj3 care).tw,kf.

6. ((make or making or maker) adj3 decision*).tw,kf.

7. (next adj3 kin).tw,kf.

8. (patient adj3 (proxy or surrogate)).tw,kf.

9. (power adj3 attorney).tw,kf.

10. critical care/ or life support care/ or advanced cardiac life support/ or advanced trauma life support care/ or terminal care/ or hospice care/ or withholding treatment/ or euthanasia, passive/ or respiration, artificial/ or critical care nursing/ or intensive care units/ or respiratory care units/

11. resuscitation/ or cardiopulmonary resuscitation/ or respiration, artificial/ or resuscitation orders/

12. patient rights/ or right to die/ or treatment refusal/

13. persistent vegetative state/ or brain death/ or brain injuries/ or exp brain hemorrhage, traumatic/ or brain injuries, traumatic/

14. Terminally Ill/

15. emergency nursing/ or "hospice and palliative care nursing"/

16. Palliative Medicine/ or palliative care/

17. (life adj3 (prolong* or sustain* or support*)).tw,kf.

18. (care adj3 (terminal or palliative or intensive or critical or hospice)).tw,kf.

19. ((ill or illness*) adj3 (terminal* or serious* or critical*)).tw,kf.

20. ICU.tw,kf.

21. Coma/

22. ((withdraw* or withhold* or refus* or discontinu* or remov* or terminat*) adj3 (treatment* or care or therap* or intervention* or medical*)).tw,kf.

23. ((end or last) adj3 life).tw,kf.

24. end of life care.mp.

25. ethnic groups/ or african americans/ or arabs/ or asian americans/ or indigenous peoples/ or inuits/ or hispanic americans/ or mexican americans/ or jews/

26. culture/ or acculturation/ or cross-cultural comparison/ or cultural characteristics/ or cultural competency/ or cultural diversity/ or minority groups/

27. religion/ or buddhism/ or exp christianity/ or hinduism/ or islam/ or judaism/ or "religion and medicine"/ or "religion and science"/

28. spirituality/

29. "Emigrants and Immigrants"/

30. (ethnic* or ethnocultur* or cultur* or race* or racial* or minorit* or religio* or spiritual* or immigra* or foreign* or divers*).tw,kf.

31. 1 or 2 or 3 or 4 or 5 or 6 or 7 or 8 or 9 [Decision Making]

32. 10 or 11 or 12 or 13 or 14 or 15 or 16 or 17 or 18 or 19 or 20 or 21 or 22 or 23 or 24 [End of Life]

33. 25 or 26 or 27 or 28 or 29 or 30 [Ethnicity/Culture]

34. 31 and 32 and 33 [Decision Making + End of Life + Ethnicity/Culture]

**Embase:**

1. decision making/ or clinical decision making/ or ethical decision making/ or family decision making/ or medical decision making/ or patient decision making/ or shared decision making/

2. patient care planning/

3. advance care planning/

4. health care planning/

5. living will/

6. informed consent/

7. decision support system/ or clinical decision support system/

8. (goal* adj3 care).tw,kw.

9. ((plan* or decision*) adj4 (care or medical or treatment)).tw,kw.

10. ((make or making or maker) adj3 decision*).tw,kw.

11. (next adj3 kin).tw,kw.

12. (patient adj3 (proxy or surrogate)).tw,kw.

13. (power adj3 attorney).tw,kw.

14. intensive care/ or artificial feeding/ or artificial ventilation/ or intensive care nursing/ or resuscitation/ or treatment withdrawal/

15. palliative therapy/

16. terminal care/ or euthanasia/ or hospice care/

17. ICU.tw,kw.

18. terminally ill patient/ or hospice patient/

19. ((care or ill or illness*) adj3 (intensive or critical* or serious*)).tw,kw.

20. intensive care unit/ or medical intensive care unit/ or neurological intensive care unit/ or stroke unit/ or surgical intensive care unit/

21. palliative nursing/ or hospice nursing/

22. right to die/

23. brain death/

24. brain damage/ or brain injury/

25. coma/

26. persistent vegetative state/

27. ((end or last) adj3 life).tw,kw.

28. (life adj3 (prolong* or sustain* or support*)).tw,kw.

29. (care adj3 (intensive or terminal or palliative or critical or hospice)).tw,kw.

30. ((ill or illness*) adj3 (terminal* or serious* or critical*)).tw,kw.

31. end of life care.mp.

32. ethnicity/

33. ethnic group/

34. exp religion/

35. minority group/ or minority health/

36. exp immigrant/ or immigration/

37. (ethnic* or ethnocultur* or cultur* or race* or racial* or minorit* or religio* or spiritual* or immigra* or foreign* or divers*).tw,kw.

38. ((withdraw* or withhold* or refus* or discontinu* or remov* or terminat*) adj3 (treatment* or care or therap* or intervention* or medical*)).tw,kw.

39. 1 or 2 or 3 or 4 or 5 or 6 or 7 or 8 or 9 or 10 or 11 or 12 or 13 [Decision Making]

40. 14 or 15 or 16 or 17 or 18 or 19 or 20 or 21 or 22 or 23 or 24 or 25 or 26 or 27 or 28 or 29 or 30 or 31 or 38 [End of Life]

41. 32 or 33 or 34 or 35 or 36 or 37 [Ethnicity/Culture]

42. 39 and 40 and 41 [Decision Making + End of Life + Ethnicity/Culture]

**PsycINFO:**

1. decision making/ or choice behavior/ or group decision making/ or critical thinking/ or decision support systems/ or judgment/ or problem solving/

2. advance directives/ or informed consent/

3. (advance adj3 plan*).tw.

4. (goal* adj3 care).tw.

5. (living adj3 will).tw.

6. ((plan* or decision*) adj4 (care or medical or treatment)).tw.

7. ((make or making or maker) adj3 decision*).tw.

8. (next adj3 kin).tw.

9. (patient adj3 (proxy or surrogate)).tw.

10. (power adj3 attorney).tw.

11. intensive care/ or life sustaining treatment/ or treatment refusal/ or treatment withholding/

12. "death and dying"/ or euthanasia/ or palliative care/ or terminally ill patients/

13. cpr/ or client rights/

14. hospice/

15. artificial respiration/

16. ((care or ill or illness*) adj3 (intensive or critical* or serious*)).tw.

17. treatment termination/

18. brain damage/ or brain death/ or brain injuries/

19. coma/

20. ((withdraw* or withhold* or refus* or discontinu* or remov* or terminat*) adj3 (treatment* or care or therap* or intervention* or medical*)).tw.

21. end of life care.mp.

22. (ill* adj3 (terminal* or serious* or critical*)).tw.

23. (care adj3 (intensive or terminal or palliative or critical or hospice)).tw.

24. right to treatment/

25. ((end or last) adj3 life).tw.

26. (life adj3 (prolong* or sustain* or support*)).tw.

27. ethnic identity/ or ethnic values/

28. religion/ or religious beliefs/ or religious experiences/ or religious groups/ or spiritual care/ or spiritual well being/ or spirituality/ or religiosity/ or religious affiliation/

29. "race and ethnic discrimination"/ or "racial and ethnic attitudes"/ or "racial and ethnic differences"/ or "racial and ethnic groups"/ or african cultural groups/ or arabs/ or asians/ or blacks/ or european cultural groups/ or indigenous populations/ or "latinos/latinas"/ or whites/ or cross cultural communication/ or cross cultural differences/ or cross cultural psychology/ or cross cultural treatment/ or cultural sensitivity/ or "culture (anthropological)"/ or ethnic values/ or majority groups/ or minority groups/ or multiculturalism/ or multiracial/ or "race (anthropological)"/ or religious groups/ or "racial and ethnic relations"/

30. (ethnic* or ethnocultur* or cultur* or race* or racial* or minorit* or religio* or spiritual* or immigra* or foreign* or divers*).tw.

31. immigration/ or human migration/ or refugees/

32. 1 or 2 or 3 or 4 or 5 or 6 or 7 or 8 or 9 or 10 [Decision Making]

33. 27 or 28 or 29 or 30 or 31 [Ethnicity/Culture]

34. 11 or 12 or 13 or 14 or 15 or 16 or 17 or 18 or 19 or 20 or 21 or 22 or 23 or 24 or 25 or 26 [End of Life]

35. 32 and 33 and 34 [Decision Making + End of Life + Ethnicity/Culture]

**CINAHL:**

|  | S45 | S42 AND S43 AND S44 [Decision Making + End of Life + Ethnicity/Culture] |
| --- | --- | --- |
|  | S44 | S36 OR S37 OR S38 OR S39 OR S40 OR S41 [Ethnicity/Culture] |
|  | S43 | S16 OR S17 OR S18 OR S19 OR S20 OR S21 OR S22 OR S23 OR S24 OR S25 OR S26 OR S27 OR S28 OR S29 OR S30 OR S31 OR S32 OR S33 OR S34 OR S35 [End of Life] |
|  | S42 | S1 OR S2 OR S3 OR S4 OR S5 OR S6 OR S7 OR S8 OR S9 OR S10 OR S11 OR S12 OR S13 OR S14 OR S15 [Decision Making] |
|  | S41 | TI (ethnic* or ethnocultur* or cultur* or race* or racial* or minorit* or religio* or spiritual* or immigrant* or foreign* or divers*) |
|  | S40 | AB (ethnic* or ethnocultur* or cultur* or race* or racial* or minorit* or religio* or spiritual* or immigrant* or foreign* or divers*) |
|  | S39 | (MH "Emigration and Immigration") OR (MH "Transients and Migrants") |
|  | S38 | (MH "Religion and Medicine") OR (MH "Religion and Religions") OR (MH "Buddhism") OR (MH "Christianity") OR (MH "Catholicism") OR (MH "Hinduism") OR (MH "Islam") OR (MH "Judaism") OR (MH "Prayer") OR (MH "Spirituality") |
|  | S37 | (MH "Anthropology, Cultural") |
|  | S36 | (MH "Culture") OR (MH "Acculturation") OR (MH "Cultural Diversity") OR (MH "Cultural Safety") OR (MH "Cultural Values") OR (MH "Ethnic Groups") OR (MH "Arabs") OR (MH "Asians+") OR (MH "Blacks") OR (MH "Hispanics") OR (MH "Inuit") OR (MH "Native Americans") OR (MH "Indigenous Peoples") OR (MH "Jews") OR (MH "Kurds") OR (MH "Whites") OR (MH "Immigrants") OR (MH "Minority Groups") |
|  | S35 | TI ((withdraw* or withhold* or refus* or discontinu* or remov* or terminat*) N3 (treatment* or care or therap* or intervention* or medical*)) |
|  | S34 | AB ((withdraw* or withhold* or refus* or discontinu* or remov* or terminat*) N3 (treatment* or care or therap* or intervention* or medical*)) |
|  | S33 | TI (care N3 (critical or terminal or intensive or hospice or intensive or palliative)) |
|  | S32 | AB (care N3 (critical or terminal or intensive or hospice or intensive or palliative)) |
|  | S31 | TI (ill or illness*) N3 (critical* or terminal* or serious*) |
|  | S30 | AB (ill or illness*) N3 (critical* or terminal* or serious*) |
|  | S29 | TI (life N3 (prolong* or sustain* or support*)) |
|  | S28 | AB (life N3 (prolong* or sustain* or support*)) |
|  | S27 | TI ((end or last) N3 life) |
|  | S26 | AB ((end or last) N3 life) |
|  | S25 | (MH "Coma") OR (MH "Minimally Conscious State") OR (MH "Persistent Vegetative State") OR (MH "Brain Injuries") OR (MH "Brain Damage, Chronic") OR (MH "Brain Death") |
|  | S24 | (MH "Treatment Termination") OR (MH "Treatment Refusal") |
|  | S23 | (MH "Right to Die") OR (MH "Patient Rights") OR (MH "Right to Life") OR (MH "Euthanasia, Passive") |
|  | S22 | AB (care or ill or illness*) N3 (intensive or critical* or serious*) |
|  | S21 | TI (care or ill or illness*) N3 (intensive or critical* or serious*) |
|  | S20 | (MH "Terminally Ill Patients") OR (MH "Hospice Patients") OR (MH "Palliative Care") OR (MH "Terminal Care") OR (MH "Hospice Care") |
|  | S19 | (MH "Intensive Care Units") OR (MH "Respiratory Care Units") OR (MH "Critically Ill Patients") OR (MH "Ventilator Patients") OR (MH "Critical Care") |
|  | S18 | (MH "Emergency Nursing") OR (MH "Trauma Nursing") OR (MH "Hospice and Palliative Nursing") |
|  | S17 | (MH "Resuscitation") OR (MH "Respiration, Artificial") OR (MH "Resuscitation, Cardiopulmonary") OR (MH "Advanced Cardiac Life Support") |
|  | S16 | (MH "Life Support Care+") OR (MH "Resuscitation Orders") OR (MH "Tracheostomy Care") |
|  | S15 | AB (power N3 attorney) |
|  | S14 | TI (power N3 attorney) |
|  | S13 | AB (patient N3 (proxy or surrogate)) |
|  | S12 | TI (patient N3 (proxy or surrogate)) |
|  | S11 | AB (next N3 kin) |
|  | S10 | TI (next N3 kin) |
|  | S9 | TI (goal* N3 care) |
|  | S8 | AB (goal* N3 care) |
|  | S7 | TI ((make or making or maker) N3 decision*). |
|  | S6 | AB ((make or making or maker) N3 decision*) |
|  | S5 | TI ((plan* or decision*) N4 (care or medical or treatment)) |
|  | S4 | AB ((plan* or decision*) N4 (care or medical or treatment)) |
|  | S3 | (MH "Patient Care Plans") OR (MH "Critical Path") |
|  | S2 | (MH "Advance Directives") OR (MH "Durable Power of Attorney") OR (MH "Living Wills") OR (MH "Resuscitation Orders") OR (MH "Consent") |
|  | S1 | (MH "Decision Making") OR (MH "Consensus") OR (MH "Decision Making, Clinical") OR (MH "Decision Making, Ethical") OR (MH "Decision Making, Family") OR (MH "Decision Making, Patient") OR (MH "Advance Care Planning") OR (MH "Decision Making, Shared") OR (MH "Decision Support Techniques") OR (MH "Dissent and Disputes") OR (MH "Refusal to Participate") OR (MH "Goal-Setting") OR (MH "Judgment") OR (MH "Problem Solving") |
