# Supplementary material for: The effect of end-of-life decision-making tools on patient and family-related outcomes of care among ethnocultural minorities: A systematic review
Source: PLoS One. 2022 Aug 4;17(8):e0272436. doi: 10.1371/journal.pone.0272436 (PMC9352046; doi:10.1371/journal.pone.0272436)
Supplement: S3 Appendix — (DOCX) [file pone.0272436.s003.docx]

S3 Appendix. Risk of bias and quality assessments of individual studies.

Table 1. Risk of bias assessment for randomized controlled trials.

**
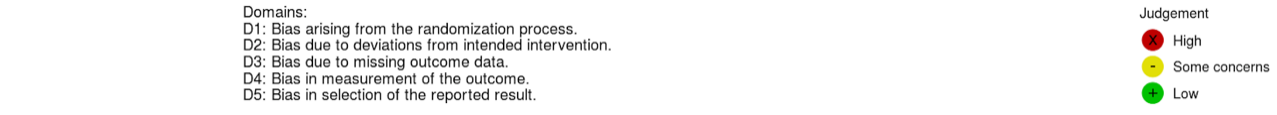

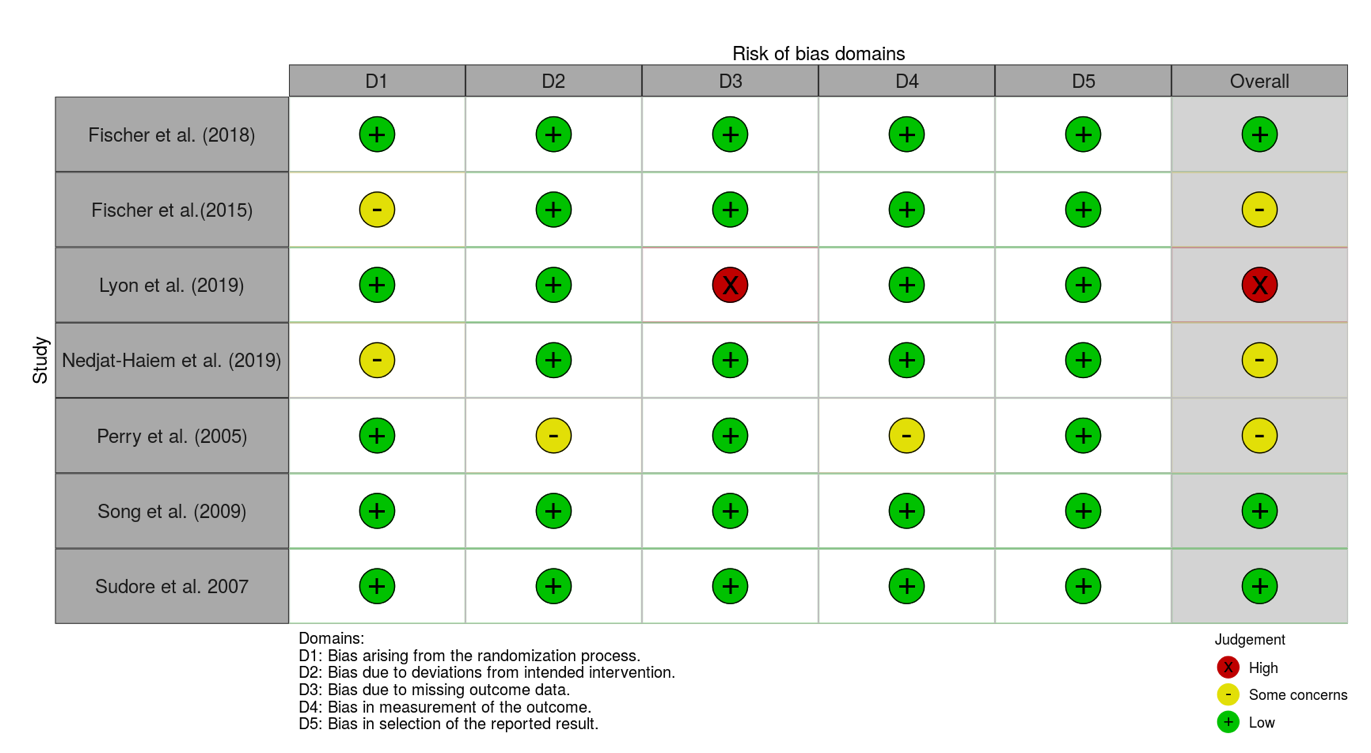
**

Table 2. Risk of bias assessment for cluster randomized controlled trials.


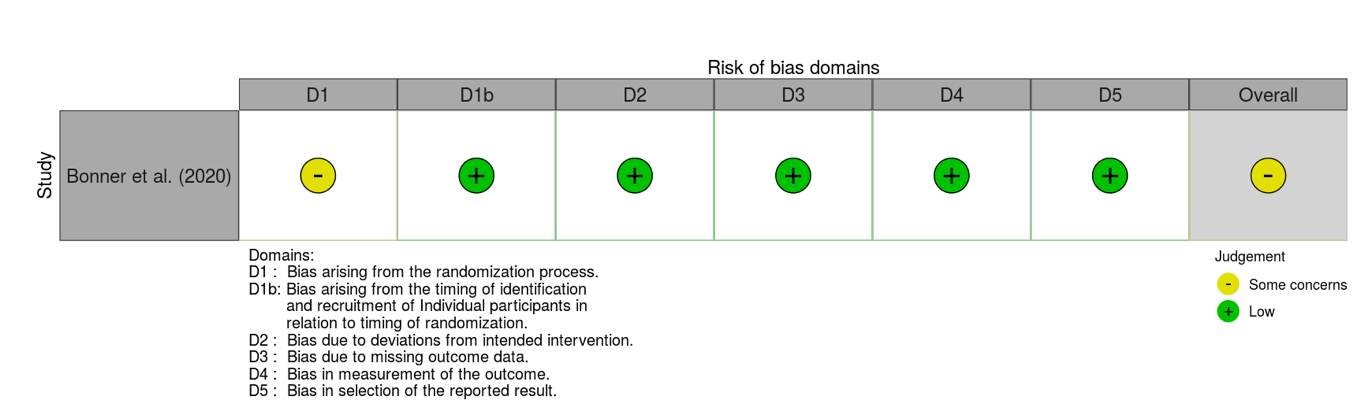


Table 3. Quality assessment of observational cohort studies.

| **Source** | **Selection** | **Comparability** | **Outcome** | **Overall Risk of Bias** |
| --- | --- | --- | --- | --- |
| Bell et al. (2011) | ★★★★ | ★★ | ★★★ | Good |
| Benton et al. (2015) | ★★★★ | ☆☆ | ★★★ | Poor |
| Gonzalez et al. (2021) | ★★★★ | ★★ | ★★★ | Good |
| Pecanac et al. (2014) | ★★★★ | ★★ | ★★★ | Good |
| Roth et al. (2020) | ★★★☆ | ☆☆ | ★★★ | Poor |
| Sacco et al. (2012) | ★★★★ | ☆☆ | ★★★ | Poor |
| Shen et al. (2016) | ★★☆☆ | ★★ | ★★☆ | Fair |
| Zaide et al. (2012) | ★★★★ | ★☆ | ★★☆ | Good |

^a^ Using the Newcastle-Ottawa Scale, stars are awarded for each quality item, with the maximum number of stars in the "Selection," "Comparability," and "Outcome" being four, two, and three, respectively. In the table, solid stars indicate stars awarded for quality items, while open stars indicate quality items which were absent.

^b^ Thresholds for converting the Newcastle-Ottawa scales to quality standards (good, fair, and poor): Good quality: 3 or 4 stars in selection domain AND 1 or 2 stars in comparability domain AND 2 or 3 stars in outcome/exposure domain; Fair quality: 2 stars in selection domain AND 1 or 2 stars in comparability domain AND 2 or 3 stars in outcome/exposure domain; Poor quality: 0 or 1 star in selection domain OR 0 stars in comparability domain OR 0 or 1 stars in outcome/exposure domain.

Table 4. Quality assessment of non-randomized interventional studies.

| Source | **Bonner**  **et al. (2014)** | **Braun**  **et al. (2006)** | **Dhingra**  **et al. (2021)** | **Fernandes**  **et al. (2010)** | **Lee**  **et al. (2015)** | **Maldonado et al. (2017)** | **Park**  **et al. (2021)** | **Patel**  **et al. (2021)** | **Radhakrishnan et al. (2019)** | **Song et al. (2010)** | **Sudore**  **et al. (2007)** | **Sudore**  **et al. (2014)** | **Sudore**  **et al. (2018)** | **Sun**  **et al. (2017)** | **Volandes et al. (2007)** | **Volandes et al. (2008)** |
| --- | --- | --- | --- | --- | --- | --- | --- | --- | --- | --- | --- | --- | --- | --- | --- | --- |
| Clearly Stated Aim | 2 | 1 | 1 | 2 | 2 | 2 | 1 | 2 | 2 | 2 | 2 | 2 | 2 | 2 | 2 | 2 |
| Inclusion of consecutive patients | 2 | 2 | 2 | 2 | 2 | 2 | 0 | 2 | 2 | 2 | 2 | 2 | 2 | 2 | 2 | 2 |
| Prospective data collection | 2 | 2 | 2 | 2 | 2 | 2 | 2 | 2 | 2 | 2 | 2 | 2 | 2 | 2 | 2 | 2 |
| Endpoints appropriate to study aim | 2 | 2 | 2 | 2 | 2 | 2 | 2 | 2 | 2 | 2 | 2 | 2 | 2 | 2 | 2 | 2 |
| Unbiased assessment of study endpoints | 2 | 2 | 1 | 1 | 2 | 2 | 2 | 2 | 1 | 2 | 2 | 2 | 2 | 2 | 2 | 2 |
| Follow up period appropriate to study aim | 2 | 2 | 2 | 2 | 2 | 2 | 2 | 2 | 2 | 2 | 2 | 1 | 2 | 2 | 2 | 1 |
| Loss to follow up <5% | 1 | 1 | 2 | 1 | 2 | 2 | 1 | 2 | 1 | 2 | 2 | 2 | 2 | 1 | 2 | 2 |
| Prospective calculation of the study size | 0 | 0 | 0 | 0 | 0 | 0 | 0 | 2 | 0 | 2 | 2 | 0 | 2 | 0 | 0 | 1 |
| Adequate control group^c^ | 2 | - | - | - | - | - | - | 2 | - | 2 | 2 | - | 2 | - | - | - |
| Contemporary groups^c^ | 2 | - | - | - | - | - | - | 2 | - | 2 | 2 | - | 2 | - | - | - |
| Baseline equivalence of groups^c^ | 2 | - | - | - | - | - | - | 2 | - | 2 | 2 | - | 2 | - | - | - |
| Adequate statistical analysis^c^ | 1 | - | - | - | - | - | - | 2 | - | 2 | 2 | - | 2 | - | - | - |
| Total Score | 20 | 12 | **12** | 12 | 14 | 14 | **10** | **24** | 12 | **24** | **24** | 13 | **24** | 13 | 14 | 14 |

^a^ The items are scored 0 (not reported), 1 (reported but inadequate) or 2 (reported and adequate).

^b^ The global ideal score is 16 for non-comparative studies and 24 for comparative studies. For noncomparative studies, the scores are as follows: 0-4, very low quality; 5-8, low quality; 9-12, moderate quality; and 13-16, high quality. For comparative studies, the scores are as follows: 0-6, very low quality; 7-12, low quality; 13-18, moderate quality; and 19-24, high quality.

^c^ Additional criteria used in the case of comparative studies.

Table 5. Quality assessment of cross-sectional studies.

| Source | **Ortiz et al. (2015)** | **Wong et al. (2021)** |
| --- | --- | --- |
| 1) Define the source of information (survey, record review) | + | + |
| 2) List inclusion and exclusion criteria for exposed and unexposed subjects (cases and controls) or refer to previous publications | + | - |
| 3) Indicate time period used for identifying patients | - | - |
| 4) Indicate whether or not subjects were consecutive if not population-based | + | + |
| 5) Indicate if evaluators of subjective components of study were masked to other aspects of the status of the participants | + | U |
| 6) Describe any assessments undertaken for quality assurance purposes (e.g., test/retest of primary outcome measurements) | - | - |
| 7) Explain any patient exclusions from analysis | + | + |
| 8) Describe how confounding was assessed and/or controlled. | + | + |
| 9) If applicable, explain how missing data were handled in the analysis | U | - |
| 10) Summarize patient response rates and completeness of data collection | + | + |
| 11) Clarify what follow-up, if any, was expected and the percentage of patients for which incomplete data or follow-up was obtained | U | + |
| Total Score | 7  (Moderate Quality) | **6**  **(Moderate Quality)** |

^a^ Quality scores are rated as yes (+), no (-) and unclear (U). Items rated as “no” or “unclear” are given a score of 0 and items rated as “yes” are given a score of 1.

^b^ Scores 0 to 3 (low quality), 4 to 7 (moderate quality), and 8 to 11 (high quality).

Table 6. Quality assessment of qualitative and mixed-methods research studies.

| Source | **Van Scoy et al. (2020)** | **Huang et al. (2016)** | **Nedjat-Haiem et al. (2017)** | **Bullock (2006)** |
| --- | --- | --- | --- | --- |
| 1. QUALITATIVE STUDIES |  | | | |
| 1.1. Is the qualitative approach appropriate to answer the research question? | Can’t tell | Can’t tell | Can’t tell | Can’t tell |
| 1.2. Are the qualitative data collection methods adequate to address the research question? | Yes | Yes | Yes | Yes |
| 1.3. Are the findings adequately derived from the data? | Yes | Yes | Yes | Yes |
| 1.4. Is the interpretation of results sufficiently substantiated by data? | Yes | Yes | Yes | Yes |
| 1.5. Is there coherence between qualitative data sources, collection, analysis and interpretation? | Yes | Yes | Yes | Yes |
| 2. RANDOMIZED CONTROLLED TRIALS |  | | | |
| 2.1. Is randomization appropriately performed? | - | Can’t tell | Can’t tell | - |
| 2.2. Are the groups comparable at baseline? | - | Yes | Yes | - |
| 2.3. Are there complete outcome data? | - | Yes | Yes | - |
| 2.4. Are outcome assessors blinded to the intervention provided? | - | Yes | No | - |
| 2.5 Did the participants adhere to the assigned intervention? | - | Yes | Yes | - |
| 3. NON-RANDOMIZED STUDIES |  | | | |
| 3.1. Are the participants representative of the target population? | Yes | - | - | - |
| 3.2. Are measurements appropriate regarding both the outcome and intervention (or exposure)? | Yes | - | - | - |
| 3.3. Are there complete outcome data? | Yes | - | - | - |
| 3.4. Are the confounders accounted for in the design and analysis? | No | - | - | - |
| 3.5. During the study period, is the intervention administered (or exposure occurred) as intended? | Yes | - | - | - |
| 5. MIXED METHODS STUDIES |  |  |  |  |
| 5.1. Is there an adequate rationale for using a mixed methods design to address the research question? | Can’t tell | No | No | - |
| 5.2. Are the different components of the study effectively integrated to answer the research question? | Yes | Yes | Yes | - |
| 5.3. Are the outputs of the integration of qualitative and quantitative components adequately interpreted? | Yes | Yes | No | - |
| 5.4. Are divergences and inconsistencies between quantitative and qualitative results adequately addressed? | Yes | No | No | - |
| 5.5. Do the different components of the study adhere to the quality criteria of each tradition of the methods involved? | Yes | Yes | Yes | - |
| Total Score | 4  (80% of quality criteria met) | 3  (60% of quality criteria met) | 2  (40% of quality criteria met) | 4  (80% of quality criteria met) |

^a^ Section 4: Quantitative Descriptive Studies was removed from this table as we did not evaluate eligible studies with this research design using the MMAT tool.
