# Supplementary material for: The effect of end-of-life decision-making tools on patient and family-related outcomes of care among ethnocultural minorities: A systematic review
Source: PLoS One. 2022 Aug 4;17(8):e0272436. doi: 10.1371/journal.pone.0272436 (PMC9352046; doi:10.1371/journal.pone.0272436)
Supplement: S1 Table — (DOCX) [file pone.0272436.s004.docx]

S1 Table. Characteristics of eligible studies.

| **Author/Year** | **Study Design** | **Tool** | **Target Population** | **Comparator** | **Target Patient Demographic** | **Healthcare Setting(s)** | **Sample Size** | **Measured Outcome(s)** | | |
| --- | --- | --- | --- | --- | --- | --- | --- | --- | --- | --- |
|  |  |  |  |  |  |  |  | **Goals of Care and ACP** | **Patient/Family Satisfaction** | **Healthcare Utilization** |
| Bell et al. (2011) | Prospective Cohort | Healthcare Provider-Led Intervention: Palliative Care Consultation | Patients | Usual Care | Multi-ethnic | Urban Hospital | Intervention n=1362 | • DNR Status | - | • Hospice Use |
| Benton et al. (2015) | Retrospective Cohort | Healthcare Provider-Led Intervention: Palliative Care Consultation | Patients | Before PCC | African American | Urban Hospital | Intervention n=2843 | • Preferences for Care | - | - |
| Bonner et al. (2014) | Two-Group Pre-Post | Education: ACT-Plan | Families | Usual Care | African American | Community | Intervention n=35, Control n=33 | • Preferences for Care | - | - |
| Bonner et al. (2020) | Cluster RCT | Education: ACT-Plan | Families | Usual Care | African American | Community | Intervention n=173, Control n=185 | • Completion of ACP/AD | - | - |
| Braun et al. (2006) | One-Group Pre-Post | Education: Educational Booklet | Families | N/A | Multi-ethnic | Community | Intervention n=424 | • Completion of ACP/AD  • Preferences for Care | • Tool Satisfaction | - |
| Bullock (2006) | Qualitative | ACP Program: Faith-Based Promotion Program | Patients | N/A | African American | Community | Intervention n=102 | • Completion of ACP/AD | - | - |
| Dhingra et al. (2021) | One-Group Pre-Post | ACP Program: Culturally-Relevant ACP Toolkit | Healthcare providers | N/A | Chinese American | Community | Intervention n=66 | • Completion of ACP/AD | - | - |
| Fernandes et al. (2010) | One-Group Pre-Post | Healthcare Provider-Led Intervention: Home-Based Palliative Care Program | Patients/  Families | N/A | Multi-ethnic | Primary Care Clinic | Intervention n=91 | • Completion of ACP/AD | • Quality of Life  • Satisfaction with Care | • Hospice Length of Stay  • Emergency Department Use  • Acute Care Admissions |
| Fischer et al. (2015) | RCT | Healthcare Provider-Led Intervention: Culturally-Tailored Patient Navigator Program | Patients | Usual Care | Latino | Urban Hospital | Intervention n=32, Control n=32 | • Completion of ACP/AD | - | • Hospice Use  • Hospice Length of Stay |
| Fischer et al. (2018) | RCT | Healthcare Provider-Led Intervention: Culturally-Tailored Patient Navigator Program | Patients | Usual Care | Latino | Outpatient Care: Cancer Clinic | Intervention n=112, Control n=111 | • Completion of ACP/AD | • Quality of Life  • Pain Management | • Hospice Length of Stay |
| Gonzalez et al. (2021) | Retrospective Cohort | Communication: Language interpretation | Patients | N/A | Latino | Community | Intervention n=15,656 | • Completion of ACP/AD | - | - |
| Huang et al. (2016) | Mixed Methods | ACP Program: Multi-component ACP program | Patients | Usual Care | African American | Community | Intervention n=15, Control n=15 | - | • Tool Satisfaction | - |
| Lee et al. (2015) | One-Group Pre-Post | Education: Culturally-Sensitive Seminar | Patients | N/A | Chinese American | Community | Intervention n=72 | • Completion of ACP/AD | - | - |
| Lyon et al. (2019) | RCT | ACP Program: Family-Centred ACP | Patients/  Families | Usual Care | African American | Outpatient Care: hospital-based HIV Clinic | Intervention n=155, Control n=68 | • Completion of ACP/AD | - | - |
| Maldonado et al. (2019) | One-Group Pre-Post | Decision Aide: Five Wishes | Patients | N/A | Latino | Primary Care Clinic | Intervention n=41 | • Completion of ACP/AD | • Tool Satisfaction | - |
| Nedjat-Haiem et al. (2017) | Mixed Methods | ACP Program: ACP-Intervention | Patients | Usual Care | Latino | Community | Intervention n=35, Control n=39 | - | • Acceptability | - |
| Nedjat-Haiem et al. (2019) | RCT | ACP Program: Motivational Interviewing | Patients | Usual Care | Latino | Community | Intervention n=39, Control n=35 | • Completion of ACP/AD | - | - |
| Ortiz and Martinez (2015) | Cross-Sectional | Communication: Survival Chance | Patients | N/A | Latino | Outpatient Care: Geriatric Clinic | Intervention n=222 | • Preferences for Care | - | - |
| Park et al. (2021) | One-Group Pre-Post | ACP Program: Faith Community Nurse Intervention | Families | N/A | Korean American | Community | Intervention n=1362 | • Completion of ACP/AD | - | - |
| Patel et al. (2021) | Two-Group Pre-Post | Healthcare Provider-Led Intervention: Lay Health Worker–Led Intervention | Patients | Usual Care | Multi-ethnic | Outpatient Care: Cancer Clinic | Intervention n=66, Control n=72 | • Completion of ACP/AD | • Quality of Life | • Hospice Use |
| Pecanac et al. (2014) | Retrospective Cohort | ACP Program: Respecting Choices | Patients | Usual Care | Multi-ethnic | Urban Hospital | Intervention n=732 | • Completion of ACP/AD  • Congruence of Care | - | - |
| Perry et al. (2005) | RCT | ACP Program: Peer Mentoring | Patients | Usual Care | African American | Outpatient Care: Dialysis Clinic | Intervention n=41, Control n=125 | • Completion of ACP/AD | • Psychological Well-Being | - |
| Radhakrishnan et al. (2019) | One-Group Pre-Post | ACP Program: End-of-Life Conversation Game (Hello) | Patients | N/A | South Asian Indian American | Community | Intervention n=47 | • Completion of ACP/AD | • Tool Satisfaction  • Quality of Communication | - |
| Roth et al. (2020) | Retrospective Cohort | ACP Program: My Life, My Way | Patients | Usual Care | Multi-ethnic | Urban Hospital | Intervention n=39,080 | • Completion of ACP/AD  • DNR status | - | - |
| Sacco et al. (2013) | Retrospective Cohort | Healthcare Provider-Led Intervention: Palliative Care Consultation | Patients | Before PCC | Multi-ethnic | Urban Hospital | Intervention n=1999 | • DNR Status | - | - |
| Shen et al. (2016) | Prospective Cohort | Communication: End-of-Life Discussion | Patients | Usual Care | Latino | Outpatient Care: Cancer Clinic | Intervention n=117 | • DNR Status | - | - |
| Song et al. (2009) | RCT | ACP Program: Sharing Patient’s Illness Representations to Increase Trust [SPIRIT] | Patients/  Families | Usual Care | African American | Outpatient Care: Dialysis Clinic | Intervention n=29, Control n=29 | • Congruence of Care | • Psychological Well-Being  • Quality of Communication  • Tool Satisfaction | - |
| Song et al. (2010) | Two-Group Pre-Post | ACP Program: Patient-Centred ACP [PC-ACP] | Patients/  Families | Usual Care | African American | Outpatient Care: Dialysis Clinic | Intervention n=11, Control n=8 | •  Preferences for Care  • Congruence of Care | • Psychological Well-being  • Quality of Communication  • Acceptability | - |
| Sudore et al. (2007) | One-Group Pre-Post | Decision Aide: Re-Designed Advance Directive | Patients | Usual Care | Multi-ethnic | Primary Care Clinic | Intervention n=103, Control=102 | • Completion of ACP/AD | • Acceptability | - |
| Sudore et al. (2014) | One-Group Pre-Post | Decision Aide: PREPARE Website | Families | N/A | Multi-ethnic | Community | Intervention n=43 | - | • Tool Satisfaction  • Psychological Well-Being | - |
| Sudore et al. (2018) | Two-Group Pre-Post | Decision Aide: PREPARE + Easy-to-Read Advance Directive | Patients | Usual Care | Latino | Primary Care Clinic | Intervention n=481, Control n=505 | • Completion of ACP/AD | - | - |
| Sun et al. (2017) | One-Group Pre-Post | ACP Program: Church-Based, Culturally-Tailored Program | Patients | N/A | Asian American | Community | Intervention n=174 | • Completion of ACP/AD | - | - |
| VanScoy et al. (2020) | Mixed Methods | ACP Program: End-of-Life Conversation Game (Hello) | Patients | N/A | African American | Community | Intervention n=220 | • Completion of ACP/AD | • Tool Satisfaction | - |
| Volandes et al. (2007) | One-Group Pre-Post | Education: Video | Patients | N/A | Multi-ethnic | Primary Care Clinic | Intervention n=120 | •  Preferences for Care | • Acceptability | - |
| Volandes et al. (2008) | One-Group Pre-Post | Education: Video | Patients | N/A | Latino | Primary Care Clinic | Intervention n=104 | •  Preferences for Care | • Acceptability | - |
| Wong et al. (2021) | Cross-Sectional | Education: ACP Co-design Education Program | Patients | N/A | Chinese Australian | Community | Intervention n=325 | • Completion of ACP/AD | • Acceptability | - |
| Zaide et al. (2012) | Retrospective Cohort | Healthcare Provider-Led Intervention: Palliative Care Consultation | Patients | Before PCC | Multi-ethnic | Urban Hospital | Intervention n=533 | • Completion of ACP/AD | - | - |
