# Supplementary material for: The effect of end-of-life decision-making tools on patient and family-related outcomes of care among ethnocultural minorities: A systematic review
Source: PLoS One. 2022 Aug 4;17(8):e0272436. doi: 10.1371/journal.pone.0272436 (PMC9352046; doi:10.1371/journal.pone.0272436)
Supplement: S2 Table — (DOCX) [file pone.0272436.s005.docx]

**S2 Table. Influence of end-of-life decision-making tools on patient and family-related outcomes of care.**

| **Ref** | **Tool** | **Goals of Care and Advance Care Planning** | | | | | **Patient and Family Satisfaction and Well-Being** | | | | | | **Healthcare Utilization** | |
| --- | --- | --- | --- | --- | --- | --- | --- | --- | --- | --- | --- | --- | --- | --- |
|  |  | *Completion of advance directives* | *Completion of DNR orders* | *Congruence in end-of-life treatment preferences* | *Consistency between patient wishes and medical orders* | *Preferences for life-prolonging treatment* | *Tool*  *acceptability* | *Tool satisfaction* | *Quality of communication* | *Perceived patient quality of life* | *Pain management and severity* | *Psychological well-being* | *Receipt of life-prolonging treatment* | *Receipt of hospice care* |
| 19 | **Advance Care Treatment Plan (ACT-Plan)**: A community-based educational intervention that is delivered by an advanced practice public health professional(s) aimed at enhancing knowledge of dementia and associated end-of-life medical treatments. | **No effect** | **-** | **-** | **-** | **-** | **-** | **-** | **-** | **-** | **-** | **-** | **-** | **-** |
| 20 | **Advance Care Treatment Plan (ACT-Plan)**: A community-based intervention delivered by an advanced practice public health professional(s) aimed at enhancing knowledge of dementia and associated end-of-life medical treatments. | **-** | **-** | **-** | **-** | **Strong** | **-** | **-** | **-** | **-** | **-** | **-** | **-** | **-** |
| 21 | **Educational booklets**: A series of booklets for family members aimed at addressing five end-of-life topics: ACP, funeral and memorial services, care for the dying, what to do when a loved one dies, and help for the bereaved. | **No effect** | **-** | **-** | **-** | **-** | **-** | **-** | **-** | **-** | **-** | **-** | **-** | **-** |
| 22 | **Faith-based ACP promotion program**: A church-based program designed to inform participants about ACP and their right to self-determination regarding their end-of-life care. | **Mild** | **-** | **-** | **-** | **-** | **-** | **-** | **-** | **-** | **-** | **-** | **-** | **-** |
| 23 | **Culturally-relevant ACP toolkit:** A toolkit of resources aimed at supporting clinicians to engage in culturally-competent communication and ACP with older first-generation Chinese Americans. | **No effect** | **Mild** | **-** | **-** | **-** | **-** | **-** | **-** | **-** | **-** | **-** | **-** | **-** |
| 24 | **Language translation:** A language translator or interpreter is provided to help guide patients and families in end-of-life conversations in their preferred language. | **Strong** | **-** | **-** | **-** | **-** | **-** | **-** | **-** | **-** | **-** | **-** | **-** | **-** |
| 25 | **Thinking Ahead**: A multicomponent ACP intervention that integrates motivational interviewing, an evidence-based ACP facilitation program (Respecting Choices), and a health-literacy adjusted advance directive. | **-** | **-** | **-** | **-** | **-** | **Mild** | **Strong** | **-** | **-** | **-** | **-** | **-** | **-** |
| 26 | **Culturally-sensitive seminar:** A culturally sensitive, 1-hour, nurse-led seminar on advance directives implemented in a Chinese community center. Participants were provided bilingual materials that included a copy of the Five Wishes tool and an advance directive form. The seminar was presented in English and translated into Chinese. | **No effect** | **-** | **-** | **-** | **-** | **-** | **-** | **-** | **-** | **-** | **-** | **-** | **-** |
| 27 | **ACP-Intervention (ACP-I):** A counselling program designed to address informational and communication needs of older Latinos. | **-** | **-** | **-** | **-** | **-** | **-** | **Strong** | **-** | **-** | **-** | **-** | **-** | **-** |
| 28 | **Motivational interviewing:** Counselling is provided by social workers to help patients cope with their chronic illness and to encourage them to engage in ACP. | **Strong** | **-** | **-** | **-** | **-** | **-** | **-** | **-** | **-** | **-** | **-** | **-** | **-** |
| 29 | **Faith community nurse intervention**: A nurse-led, church-based ACP training program aimed at preparing adult children of older adult Korean Americans to initiate ACP conversations within their families. | **No effect** | **-** | **-** | **-** | **-** | **-** | **-** | **-** | **-** | **-** | **-** | **-** | **-** |
| 30 | **End-of-life conversation game (Hello):** A game that enables participants to engage in substantive and meaningful conversations about life values and end-of-life wishes. | **No effect** | **-** | **-** | **-** | **-** | **-** | **Moderate** | **-** | **-** | **-** | **-** | **-** | **-** |
| 31 | **PREPARE website**: five overarching steps in PREPARE to help patients prepare for ACP and medical decision making: | **-** | **-** | **-** | **-** | **-** | **Strong** | **Strong** | **-** | **-** | **-** | **Mild** | **-** | **-** |
| 32 | **Church-based, culturally-tailored program**: The first session included a spiritually-based endorsement of advance directives by a church leader followed by a physician explaining its purpose and use. The second session provided instructions for completing advance directives. | **Strong** | **-** | **-** | **-** | **-** | **-** | **-** | **-** | **-** | **-** | **-** | **-** | **-** |
| 33 | **End-of-life conversation game (Hello):** A game that enables participants to engage in substantive and meaningful conversations about life values and end-of-life wishes. | **Mild** | **-** | **-** | **-** | **-** | **-** | **Strong** | **Strong** | **-** | **-** | **-** | **-** | **-** |
| 34 | **Co-designed ACP education toolkit**: A toolkit comprised of four tools: 1) a 5-minute educational video on ACP; 2) information booklet on ACP written in lay language; 3) a structured oral presentation on current ACP legislation and how to complete advance directives; 4) an adaptation of the legal advance directive. | **Mild** | **-** | **-** | **-** | **-** | **Moderate** | **-** | **-** | **-** | **-** | **-** | **-** | **-** |
| 35 | **Culturally-tailored patient navigator program**: Patients received at least five home visits from a patient navigator and a culturally tailored educational packet of written information about ACP, pain management, hospice care, and a re-designed advance directive. | **Strong** | **-** | **-** | **-** | **-** | **-** | **-** | **-** | **No effect** | **No effect** | **-** | **-** | **No effect** |
| 36 | **FAmily-CEntered ACP (FACE ACP):** Session 1: *Respecting Choices* goals of care conversation between surrogates and loved ones. Session 2: completion of *Five Wishes* advance directive tool to confirm the patient’s surrogate decision-maker and end-of-life treatment preferences. | **Strong** | **-** | **-** | **-** | **-** | **-** | **-** | **-** | **-** | **-** | **-** | **-** | **-** |
| 37 | **Communicating survival chance**: A presentation of end-of-life choices with a clause of low probability of survival to support patients and families in end-of-life decision-making. | **-** | **-** | **-** | **-** | **Strong** | **-** | **-** | **-** | **-** | **-** | **-** | **-** | **-** |
| 38 | **Lay health worker–led intervention**: A multi-component intervention led by two Health Advocates that focuses on goals of care conversations, symptom screening, and clinical trial participation. | **Strong** | **-** | **-** | **-** | **-** | **-** | **-** | **-** | **Strong** | **-** | **-** | **-** | **Strong** |
| 39 | **Peer-mentoring intervention**: Selected patients/peers are trained to support other long-term dialysis patients and empower them to engage in end-of-life care planning. | **Strong** | **-** | **-** | **-** | **-** | **-** | **-** | **-** | **-** | **-** | **Strong** | **-** | **-** |
| 40 | **End-of-life discussion**: A conversation led by physicians with patients and families around end-of-life wishes and preferences for medical care and treatment. | **-** | **Strong** | **-** | **-** | **-** | **-** | **-** | **-** | **-** | **-** | **-** | **-** | **-** |
| 41 | **Sharing Patient’s Illness Representations to Increase Trust [SPIRIT]:** A one-hour, intervention that is delivered by a trained nurse interventionist to prepare dialysis patients and their families for care at the end of life. | **-** | **-** | **Moderate** | **-** | **-** | **Moderate** | **-** | **Strong** | **-** | **-** | **Mild** | **-** | **-** |
| 42 | **Patient-Centred ACP (PC-ACP):** A one-hour session with a trained nurse interventionist that addresses five elements: representational assessment of participants' beliefs about their illness condition; exploration of gaps or misunderstandings regarding chronic kidney disease and life-sustaining treatment; creation of conditions for conceptual change; introduction of replacement information; and summarization of the discussion. | **-** | **-** | **Strong** | **-** | **Strong** | **-** | **-** | **Strong** | **-** | **-** | **No effect** | **-** | **-** |
| 43 | **In-patient plan-of-care consultations:** A prolonged discussion with patients and families by the medical team to clarify goals of care, disposition, and the appropriate use of medical interventions. | **-** | **Strong** | **-** | **-** | **-** | **-** | **-** | **-** | **-** | **-** | **-** | **-** | **Strong** |
| 44 | **Counselor-based palliative care consultation:** A clinical assessment and family conference with a palliative clinician (physician, nurse practitioner, physician assistant, or clinical nurse specialist) and a counselor from the palliative care team. | **-** | **-** | **-** | **-** | **Strong** | **-** | **-** | **-** | **-** | **-** | **-** | **-** | **-** |
| 45 | **Culturally-tailored patient navigator program**: Patients received at least five home visits from a patient navigator and a culturally-tailored educational packet of written information about ACP, pain management, hospice care, and a re-designed advance directive. | **Mild** | **-** | **-** | **-** | **-** | **-** | **-** | **-** | **-** | **-** | **-** | **-** | **No effect** |
| 46 | **Respecting Choices**: A multi-component program that is comprised of patient education materials about advance directives; training for facilitators; standardization of policies for maintaining, documenting, and using advance directives; and performance improvement processes. | **Strong** | **-** | **-** | **Strong** | **-** | **-** | **-** | **-** | **-** | **-** | **-** | **-** | **-** |
| 47 | **My Life, My Way:** A quality improvement program that provided two educational brochures (a conversation guide and ACP forms) for patients and families. A five-minute animated video was also made available on the hospital website. | **Strong** | **No effect** | **-** | **-** | **-** | **-** | **-** | **-** | **-** | **-** | **-** | **-** | **-** |
| 48 | **Palliative medicine consultation:** A multi-component consultation focused on: patient/family-centered communication; comfort and supportive interventions; education related to the disease process, prognosis, and the meaning of advanced directives; counseling on documenting DNR status; and assuring concordance between caregiver interventions and patient preferences for care. | **-** | **Strong** | **-** | **-** | **-** | **-** | **-** | **-** | **-** | **-** | **-** | **-** | **-** |
| 49 | **In-patient palliative care consultation:** A patient assessment completed by the Palliative Medicine Consultation team that focuses on physical, financial, psychosocial, and spiritual distress. | **Moderate** | **-** | **-** | **-** | **-** | **-** | **-** | **-** | **-** | **-** | **-** | **-** | **-** |
| 50 | **Home-based palliative care program**: A program delivered by a multi-disciplinary team that provides culturally-sensitive palliative care to homebound patients with advanced chronic illnesses. | **Strong** | **-** | **-** | **-** | **-** | **-** | **Strong** | **-** | **Mild** | **Moderate** | **-** | **Strong** | **Mild** |
| 51 | **Five Wishes**: A user-friendly advance care planning tool that facilitates a guided conversation about end-of-life care, including aspects of care such as comfort, dignity, and spirituality. | **Strong** | **-** | **-** | **-** | **-** | **-** | **-** | **-** | **-** | **-** | **-** | **-** | **-** |
| 52 | **Re-designed advance directive:** An advance directive re-designed to meet the needs of patients with limited literacy that is written at a fifth-grade reading level and includes culturally diverse, text-enhancing graphics. | **Strong** | **-** | **-** | **-** | **-** | **Strong** | **-** | **-** | **-** | **-** | **-** | **-** | **-** |
| 53 | **PREPARE website + easy-to-read advance directive**: A patient-directed, interactive, online ACP platform combined with an easy-to-read advance directive to document end-of-life care plans. | **Strong** | **-** | **-** | **-** | **-** | **-** | **-** | **-** | **-** | **-** | **-** | **-** | **-** |
| 54 | **Educational video on advanced dementia:** A 2-minute educational video that depicts salient features of advanced dementia to support patients and families to engage in ACP. | **-** | **-** | **-** | **-** | **Strong** | **Strong** | **-** | **-** | **-** | **-** | **-** | **-** | **-** |
| 55 | **Educational video on advanced dementia:** A 2-minute educational video that depicts salient features of advanced dementia to support patients and families to engage in ACP. | **-** | **-** | **-** | **-** | **Strong** | **Moderate** | **-** | **-** | **-** | **-** | **-** | **-** | **-** |

^a^ The strength of influence of tools on patient and family-related outcomes of care were determined based on unique outcomes from each individual study.
